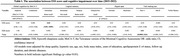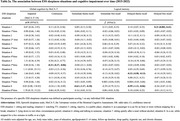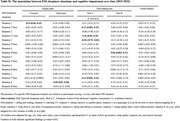# The Association between Excessive Daytime Sleepiness and Cognitive Impairment in Older Adults

**DOI:** 10.1002/alz70860_099231

**Published:** 2025-12-23

**Authors:** Cheng‐Yun Hsu, Yen‐Ching Chen, Jeng‐Min Chiou, Jen‐Hau Chen

**Affiliations:** ^1^ Institute of Epidemiology and Preventive Medicine, College of Public Health, National Taiwan University, Taipei, Taiwan; ^2^ Institute of Statistics and Data Science, National Taiwan University, Taipei, Taiwan; ^3^ Institute of Statistical Science, Academia Sinica, Taipei, Taiwan; ^4^ National Taiwan University Hospital Yunlin Branch, Yunlin, Taiwan; ^5^ College of Medicine, National Taiwan University, Taipei, Taiwan

## Abstract

**Background:**

Previous research has focused on the relationship between excessive daytime sleepiness (EDS) and the risk of developing dementia. However, limited evidence investigates the effects of EDS on domain‐specific cognitive functions or the associations between various sleepiness situations from the Epworth Sleepiness Scale (ESS) and cognitive performance. This study aims to examine the relationships between EDS, different ESS sleepiness situations, and cognitive function in community‐dwelling older adults.

**Method:**

This study (2015‐2022) included 377 non‐demented older adults from the Taiwan Initiative for Geriatric Epidemiological Research, with biennial follow‐up since 2011. Cognitive function was assessed repeatedly using the Taiwanese version of the Montreal Cognitive Assessment for global cognition and neuropsychological tests for domain‐specific cognition, including memory, attention, executive function, and language. EDS was measured by the ESS at baseline (2015‐2017) with a total score of 24 and a cutoff point of 11. The presence of a specific ESS sleepiness situation was defined as participants scoring 1 on any individual ESS situation. A generalized linear mixed model was applied to investigate the associations between EDS and cognitive performance.

**Result:**

Participants had a mean age of 75.7 and 55.2% of women. We found that one unit increment of baseline ESS score at baseline was associated with poorer performance of memory (β: ‐0.03, 95% CI: ‐0.05, ‐0.001) but associated with better performance of executive functions (β: 0.01, 95% CI: 0.0005, 0.03) over time. Regarding specific ESS sleepiness situations, over time, dozing while lying down to rest was associated with poorer global cognition (β: ‐0.21, 95% CI: ‐0.37, ‐0.06), and dozing when sitting and talking to someone was associated with poorer memory (β: ‐0.32, 95% CI: ‐0.50, ‐0.13). Conversely, dozing while sitting and reading, watching television, sitting quietly after a meal without alcohol, or as a passenger in a car for an hour or more without stopping was associated with better executive function (β: 0.06 to 0.16) over time. No significant association was found between the existence of EDS and cognition over time.

**Conclusion:**

EDS and specific sleepiness situations demonstrated mixed associations with cognition, including poor performance of global cognition and memory but improved performance of executive function over time.